# Supplementary material for: Dynamics of agonist-evoked opioid receptor activation revealed by FRET- and BRET-based opioid receptor conformation sensors
Source: Commun Biol. 2025 Feb 8;8:198. doi: 10.1038/s42003-025-07630-x (PMC11806106; doi:10.1038/s42003-025-07630-x)
Supplement: Supplementary file 1 — Supplementary Information [file 42003_2025_7630_MOESM1_ESM.pdf]

Supplemental Information

Dynamics of agonist-evoked opioid receptor activation revealed by FRET- and BRET-based opioid receptor conformation sensors

Sina B. Kirchhofer[1], Claudia Kurz[1], Lorenz Geier[1], Anna-Lena Krett[1], Cornelius Krasel[1], Moritz Bünemann[1*]

Affiliations:

[1] Department of Pharmacology and Clinical Pharmacy, University of Marburg, Karl-von-Frisch-Str. 2, 35043 Marburg, Germany

* Corresponding author Email: moritz.buenemann@staff.uni-marburg.de

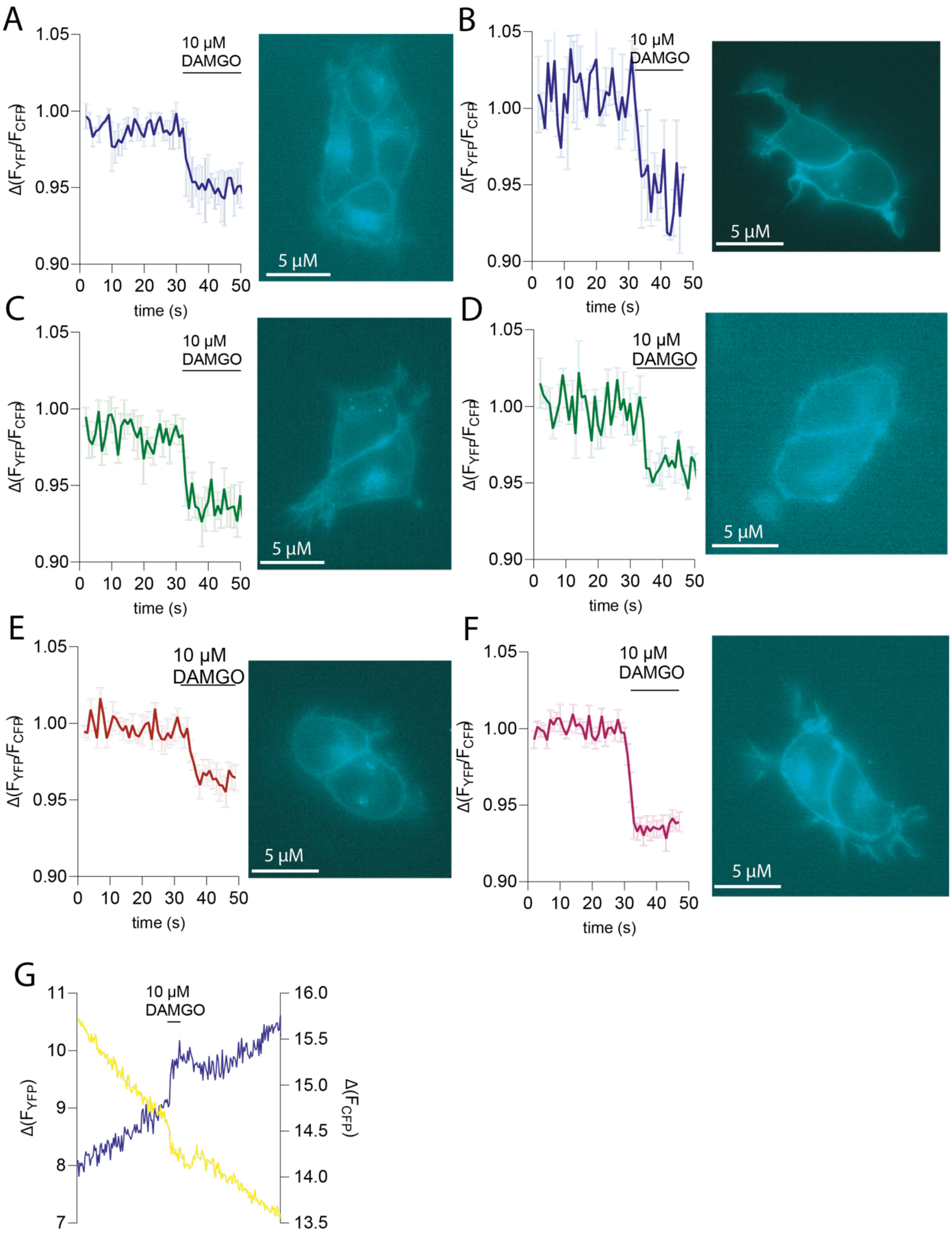

Supplemental Fig. 1: FRET Sensor constructs; A Left: Averaged FRET-based single cell recording of the MOR conformation sensor construct with sYFP inserted after M264 and mTurq2 inserted after T370. Application of DAMGO induced a decrease in FRET emission ratio by about 4% (mean ± SEM; n=12). Right: Representative picture of

HEK293 cells expressing the sensor construct in the CFP channel. **B** Left: Averaged FRET-based single cell recording of the MOR conformation sensor construct with sYFP inserted after M264 and mTurq2 inserted after T364 (M264 short). Application of DAMGO induced a decrease in FRET emission ratio by about 4% (mean ± SEM; n=2). Right: Representative picture of HEK293 cells expressing the sensor construct in the CFP channel. **C** Left: Averaged FRET-based single cell recording of the MOR conformation sensor construct with sYFP inserted after L265 and mTurq2 inserted after T370. Application of DAMGO induced a decrease in FRET emission ratio by about 4.5% (mean ± SEM; n=26). Right: Representative picture of HEK293 cells expressing the sensor construct in the CFP channel. **D** Left: Averaged FRET-based single cell recording of the MOR conformation sensor construct with sYFP inserted after L265 and mTurq2 inserted after T364 (L265 short). Application of DAMGO induced a decrease in FRET emission ratio by about 4% (mean ± SEM; n=3). Right: Representative picture of HEK293 cells expressing the sensor construct in the CFP channel. **E** Left: Averaged FRET-based single cell recording of the MOR conformation sensor construct with sYFP inserted after S266 and mTurq2 inserted after T370. Application of DAMGO induced a decrease in FRET emission ratio by about 4% (mean ± SEM; n=11). Right: Representative picture of HEK293 cells expressing the sensor construct in the CFP channel. **F** Left: Averaged FRET-based single cell recording of the MOR conformation sensor construct with sYFP inserted after G267 and mTurq2 inserted after T364 (G267 short). Application of DAMGO induced a decrease in FRET emission ratio by about 6% (mean ± SEM; n=10). Right: Representative picture of HEK293 cells expressing the sensor construct in the CFP channel. **G** Representative single fluorescence traces of a measurement of the MOR sensor G267 (Fig. 1C). Application of DAMGO induced an increase in CFP emission and a decrease in YFP emission.

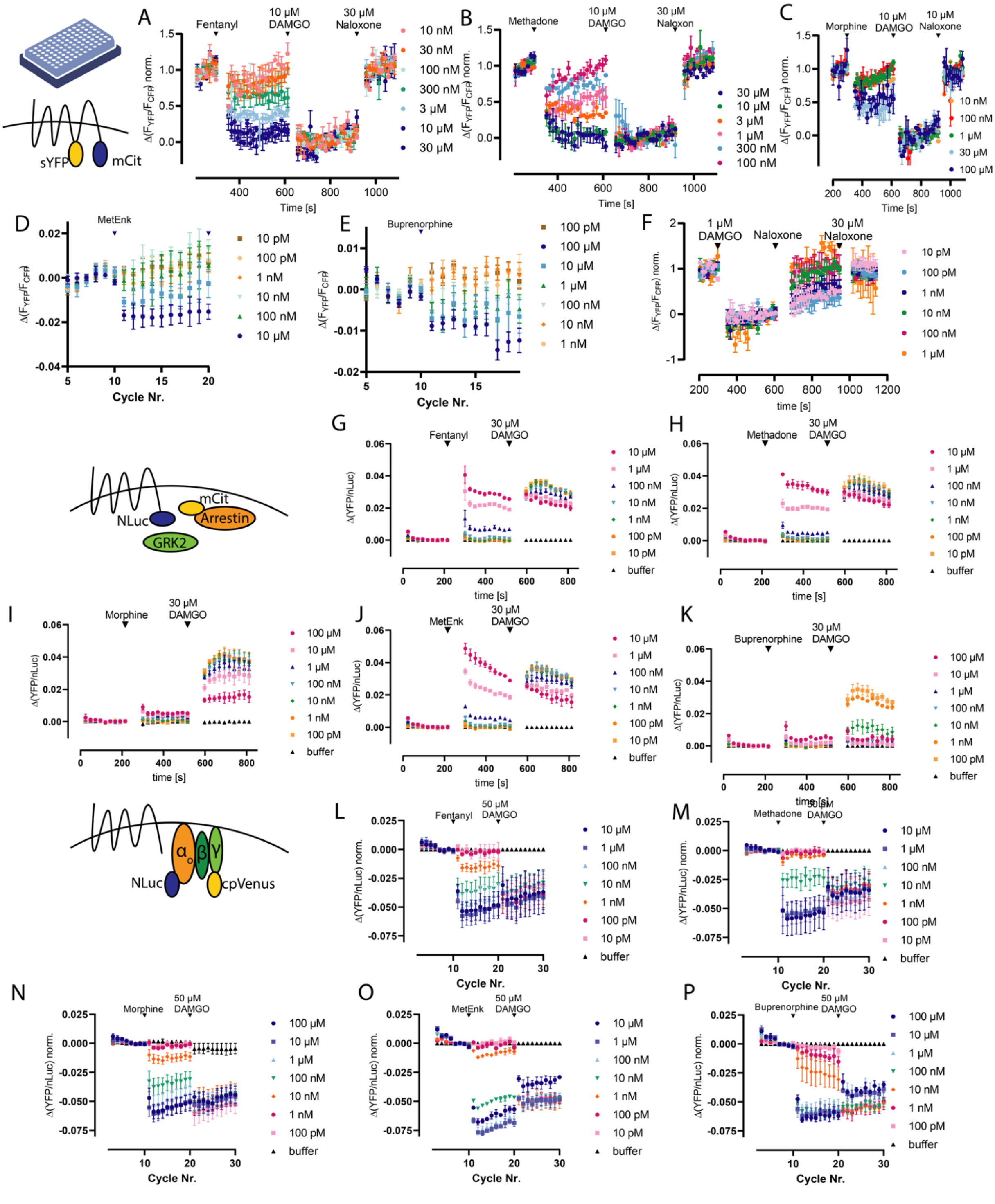

Supplemental Fig. 2 Measurements for dose response curves A-C Averaged FRET-based measurement of HEK293 cells stably expressing the MOR conformation sensor measured in 96-well plate format at a plate reader. Increasing concentrations of fentanyl (A), methadone (B) or morphine (C) were applied followed by a saturating concentration of DAMGO and the antagonist naloxone. The agonist-induced activation was normalized

to the maximal activation and plotted as concentration-response curve (Fig. 2B) (mean ± SEM, n=3 of independent transfections measured in triplets). **D-E** Averaged FRET-based measurement of HEK293 cells stably expressing the MOR conformation sensor measured in 96-well plate format at a plate reader using increasing concentrations of Met-enkephalin (D) or buprenorphine (E). The agonist-induced activation was normalized to the max. activation and plotted as concentration response curve (Fig. 2B) (mean ± SEM, n=3 of independent transfections measured in triplets). **F** Averaged FRET-based measurement of HEK293 cells stably expressing MOR conformation sensor measured in 96-well plate format at a plate reader. 1 µM DAMGO was applied and the activation was inhibited by increasing concentrations of naloxone, followed by the application of 30 µM naloxone. The antagonist-induced deactivation was normalized to the max. deactivation and plotted as inhibition curve (Fig. 2C) (mean ± SEM, n=3 of independent transfections measured in triplets). **G-K** Averaged BRET-based measurement of HEK293T cells expressing MOR-nLuc, sYFP-Arrestin and GRK. Increasing concentrations of the respective agonist were applied followed by a saturating concentration of DAMGO. The agonist-induced activation was normalized to the max. activation and plotted as concentration response curve (Fig. 2E) (mean ± SEM, n=3 of independent transfections measured in triplets). **L-P** Averaged BRET-based measurement of HEK293T cells expressing MOR and the Go-case BRET-sensor. Increasing concentrations of the respective agonist were applied followed by a saturating concentration of DAMGO. The agonist-induced activation was normalized to the max. activation and plotted as concentration response curve (Fig. 2G) (mean ± SEM, n=3 of independent transfections measured in triplets).

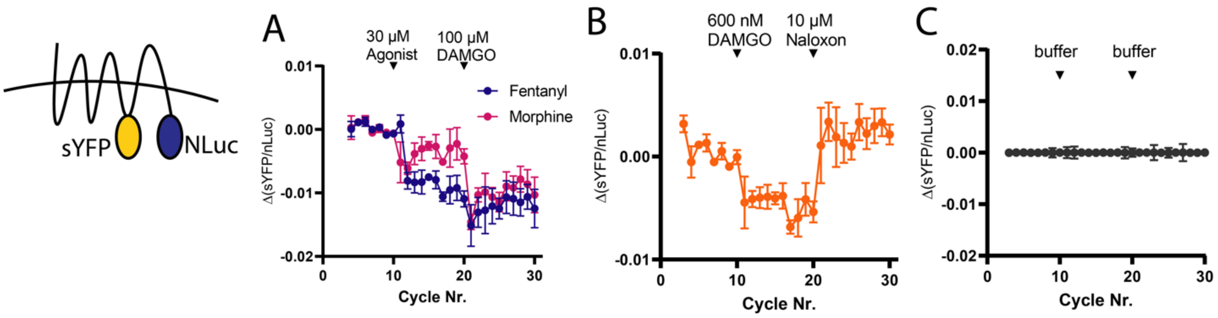

Supplemental Fig. 3 Control measurements for the BRET based sensor. A Averaged BRET-based measurement of HEK293 cells stably expressing the MOR BRET-based conformation sensor measured in 96-well plate format at a plate reader. Fentanyl (blue) or morphine (magenta) were applied followed by a saturating concentration of DAMGO (mean ± SEM, n=3 of independent transfections measured in triplets). **B** Averaged BRET-based measurement of HEK293 cells stably expressing the MOR BRET-based conformation sensor. A non-saturating concentration of DAMGO was applied followed by the application of the antagonist naloxone (mean ± SEM, n=3 of independent transfections measured in triplets). **C** Averaged BRET-based measurement of HEK293 cells stably expressing the MOR BRET-based conformation sensor. As control, extracellular buffer solution was applied two times, inducing no change in BRET ratio (mean ± SEM, n=3 of independent transfections measured in triplets).

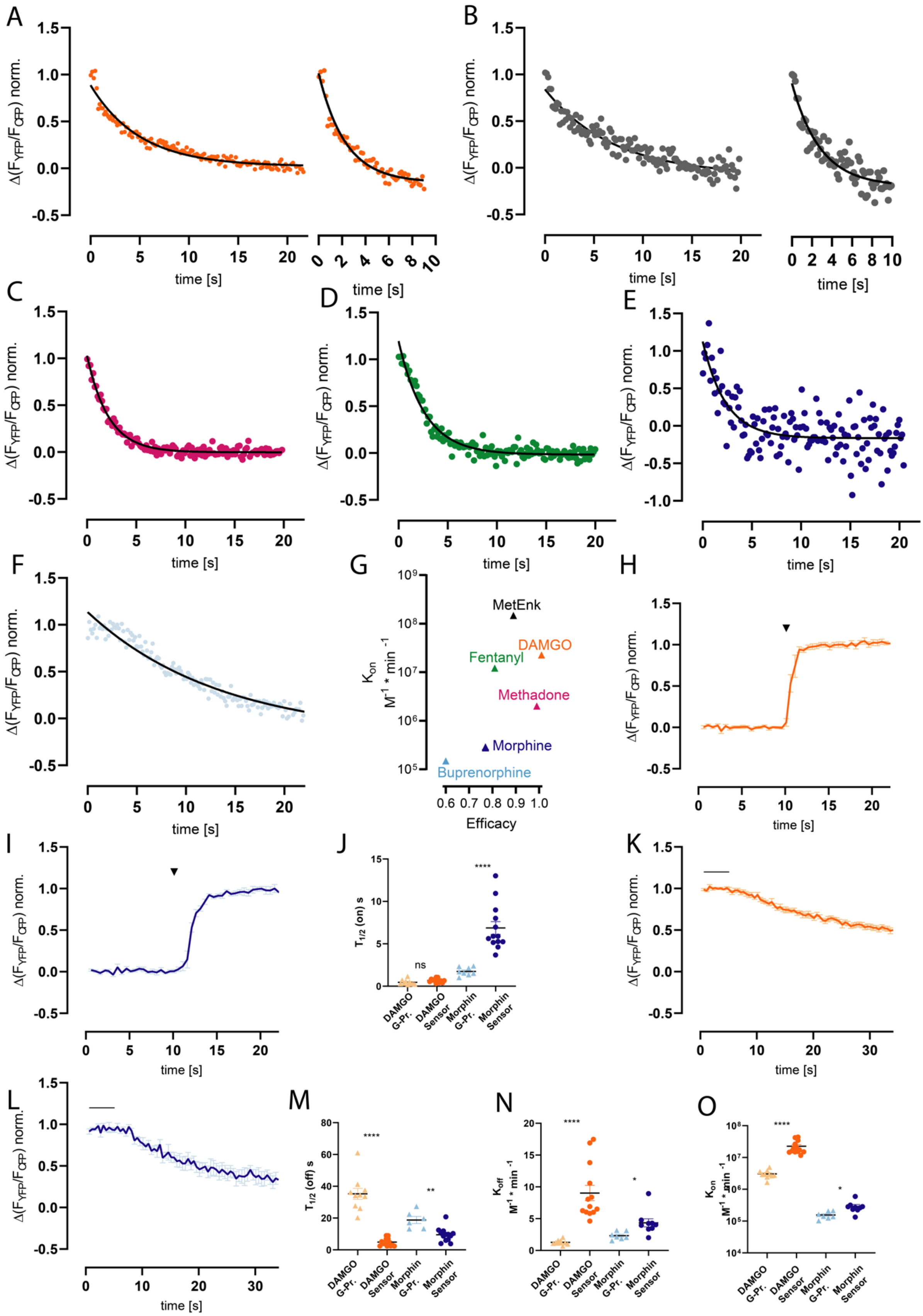

Supplemental Fig. 4: Activation kinetics of the MOR. A-F Representative single-cell FRET-based measurement of HEK293 cells stably expressing the MOR conformation sensor. Measurements were recorded with a frequency of 10 Hz and cells were

superfused with 100 µM of DAMGO (A), Met-enkephalin (B), methadone (C), fentanyl (D), morphine (E) or buprenorphine (F). A one phase mono-exponential fit was applied, shown in black. As DAMGO (A) and Met-Enkephalin (B) displayed a faster on-rate than the other agonists, the fit did not reflect all data points in the first second. To not exclude the data points in the first second, we shortened the time frame for the fit to 10 s (A and B, right panel), now including also the first data points. **G** The calculated K_{on} was plotted against the efficacy of the respective agonist indicated by the E_{max} obtained in Fig. 2B. **H-I** Averaged single-cell FRET-based measurement of HEK293T cells expressing MORsYFP, Gαo, Gβ and mTurq-Gγ. Cells were superfused with 10 µM of DAMGO (G, n=) or 30 µM of morphine (H, n=). **J** Half-time of activation was evaluated by a one phase mono-exponential fit based on G and H and compared to Fig. 4C (unpaired students t-test). **K-L** agonist wash-out of the data shown in G and H, wash-out of DAMGO (J) and morphine (K). **M** Half-time of deactivation was evaluated by a one phase mono-exponential fit based on J and K and compared to Fig. 4D (unpaired students t-test). **N** Kinetics of deactivation were analyzed by a one phase mono-exponential fit and compared to Fig. 4E (unpaired students t-test). **O** Kinetics of activation was calculated based on the kinetics of deactivation (M and Fig. 4E) and the EC_{50} of the respective agonist (Table 1); (unpaired students t-test).

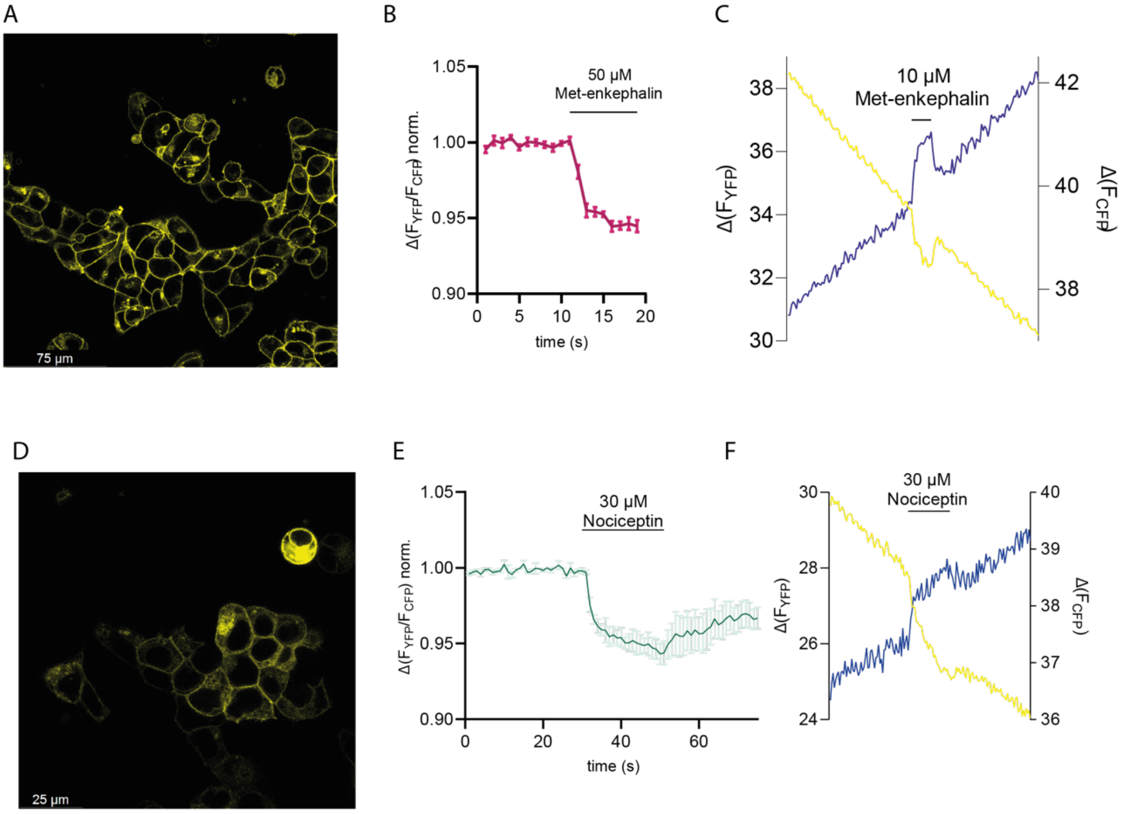

Supplemental Fig. 5: FRET Sensor constructs for the KOR and NOP; A Representative picture of HEK293 cells expressing the KOR FRET sensor construct in the YFP channel. **B** Averaged FRET-based single cell recording of the KOR conformation sensor. Application of Met-Enkephalin induced a decrease in FRET emission ratio by about 6% (mean ± SEM; n=6). **C** Representative single fluorescence traces of a measurement of the KOR sensor. Application of Met-Enkephalin induced an increase in CFP emission and a decrease in YFP emission. **D** Representative picture of HEK293 cells expressing the NOP FRET sensor construct in the YFP channel. **E** Averaged FRET-based single cell recording of the NOP conformation sensor. Application of nociception induced a decrease in FRET emission ratio by about 6% (mean ± SEM; n=6). **F** Representative single fluorescence traces of a measurement of the NOP sensor. Application of nociceptin induced an increase in CFP emission and a decrease in YFP emission.
